# Women’s Health Wearables: From Continuous Signals to Actionable Digital Phenotypes Across the Reproductive Lifespan

**DOI:** 10.3390/bioengineering13080897

**Published:** 2026-08-05

**Authors:** Rawan AlSaad, Georgianna Lin, Shima Albasha, Sara Kashani, Rajat Thomas

**Affiliations:** 1 AI Center for Precision Health, Weill Cornell Medicine-Qatar, Doha P.O. Box 24144, Qatar; 2 Department of Biomedical Informatics, Columbia University, New York, NY 10032, USA; 3 Women’s Wellness and Research Center, Hamad Medical Corporation, Doha P.O. Box 3050, Qatar; 4 College of Medicine, University of Illinois Chicago, Chicago, IL 60612, USA

**Keywords:** women’s health, wearable, digital phenotyping, pregnancy, postpartum, menopause, menstrual health, reproductive health, artificial intelligence

## Abstract

Wearable technologies are reshaping women’s health by extending observation beyond episodic clinical encounters into daily life. Across the reproductive lifespan, they can capture physiological, behavioral, symptom, and functional trajectories that are often missed in routine care. Yet more data do not automatically translate into better care. Clinical value depends on whether multimodal signals can be modeled and interpreted in relation to reproductive biology, temporal change, and meaningful clinical or functional endpoints. In this perspective, we examine how women’s health wearables can move beyond consumer tracking toward validated digital phenotyping across menstruation, fertility, pregnancy, postpartum recovery, and menopause. We propose a four-layer framework spanning data capture, physiological domain mapping, computational phenotyping, and actionable translation. We then apply this framework across key reproductive life stages. Menstrual health and fertility applications illustrate the shift from calendar-based prediction toward physiological, metabolic, and hormone-aware monitoring. Pregnancy and postpartum applications highlight the need for safety-focused validation, maternal–infant risk awareness, and clinician-governed escalation pathways. Menopause and midlife health represent underdeveloped areas where longitudinal digital phenotyping may better capture vasomotor, sleep, mood, fatigue, and functional symptoms. Across these domains, we identify key barriers to translation, including limited hormone-linked validation, inconsistent evidence standards, underrepresentation of diverse populations, privacy risks, algorithmic bias, and weak workflow integration. By organizing wearable-derived signals across reproductive life stages and identifying major translational barriers, this perspective provides a roadmap toward biologically grounded, equitable, and clinically actionable digital phenotyping for women’s health.

## 1. Introduction: Why Women’s Health Wearables Matter Now

Wearable technologies are expanding the measurement boundaries of women’s health by enabling continuous, real-world capture of physiology, behavior, symptoms, and function beyond conventional clinical settings. Although initially developed and marketed largely as consumer tools for activity tracking, sleep summaries, and menstrual logging, these technologies are increasingly being adapted to capture longitudinal signals relevant to reproductive and life-course health [1]. When interpreted within an appropriate biological and clinical context, such data may provide insight into menstrual physiology, fertility, pregnancy adaptation, postpartum recovery, perimenopause, menopause, and later-life functional health.

Several converging shifts distinguish contemporary women’s health wearables from earlier menstrual calendars and generic fitness trackers. The field is moving from manual logging and single-device tracking toward passive, multimodal sensing, personalized baselines, and clinically interpretable life-course monitoring. Current technologies include rings and wrist-worn devices that capture heart rate, HRV, peripheral temperature, sleep, activity, and respiratory estimates; physiological patches measuring ECG, temperature, respiration, and movement; continuous glucose monitors; and fertility or hormone-linked systems incorporating urinary hormones, temperature, and cycle-related symptoms [1]. These tools support menstrual and fertility tracking, pregnancy and postpartum monitoring, gestational diabetes management, and menopause symptom assessment, but remain limited by proprietary algorithms, indirect physiological proxies, variable adherence and sensor performance, heterogeneous reference standards, cost and access barriers, and limited validation in diverse populations.

This evolution is important because many aspects of women’s health remain under-measured in routine care. Episodic clinical encounters, retrospective symptom recall, calendar-based estimates, and intermittent laboratory testing are often poorly suited to conditions and transitions that fluctuate over days, cycles, trimesters, months, or years. These include menstrual symptoms, ovulatory changes, pregnancy adaptation, postpartum recovery, vasomotor symptoms, sleep disruption, and menopausal mood vulnerability. Wearables offer a complementary measurement paradigm by enabling repeated observation of physiology and behavior in daily life. For example, recent studies have shown that wearable-derived cardiovascular signals vary systematically across the menstrual cycle, supporting the potential of longitudinal heart rate and heart rate variability patterns as non-invasive markers of reproductive physiology [2].

However, expanded measurement should not be equated with clinical readiness. Greater data volume does not automatically translate into better care, and wearable-derived outputs are only useful when they are technically reliable, biologically plausible, clinically validated, interpretable, equitable, and actionable. The central challenge is therefore not whether women can collect more data, but whether these data can be transformed into trustworthy digital phenotypes that improve understanding, monitoring, and decision-making across the reproductive lifespan.

In this perspective, we examine the evolving role of women’s health wearables as they move from consumer wellness tools toward wearable-centered digital phenotyping ecosystems, including AI-enabled approaches when appropriate. We define a conceptual framework for translating wearable data into women’s health digital phenotypes; review key applications across menstruation, fertility, pregnancy, postpartum recovery, perimenopause, and menopause; and evaluate the evidence standards needed to distinguish wellness insights from clinically actionable outputs. We also address governance, equity, privacy, and implementation challenges that must be resolved for responsible integration into women’s healthcare. Together, these elements provide a translational roadmap across the reproductive lifespan.

This Perspective was informed by a targeted narrative search of PubMed, Scopus, and Google Scholar for English-language literature published primarily between 2015 and 2026, supplemented by earlier foundational studies and relevant professional and regulatory guidance. Search concepts combined women’s health and wearable or digital phenotyping terms with menstruation, fertility, pregnancy, postpartum, menopause, artificial intelligence, validation, privacy, equity, and implementation. Evidence was selected purposively to represent major reproductive life stages, signal modalities, validation frameworks, and translational challenges, with preference given to recent empirical studies, authoritative guidance, and studies providing biological or clinical validation. This approach was not systematic or exhaustive but was designed to support the conceptual and translational aims of this Perspective.

## 2. A Four-Layer Framework for Translating Wearable Signals into Women’s Health Digital Phenotypes

**Figure 1:**
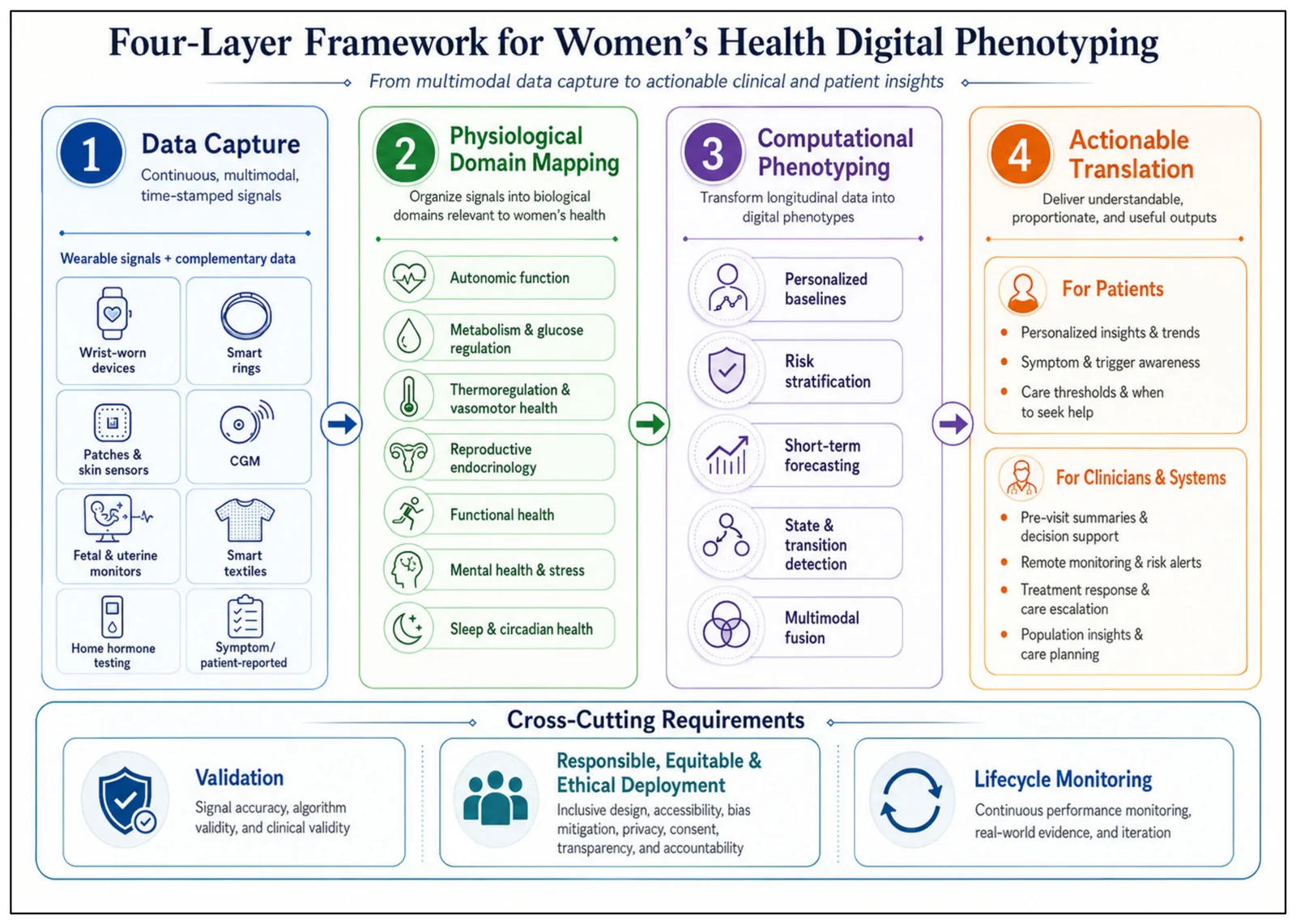
Four-layer framework for women’s health digital phenotyping. The framework illustrates how wearable signals and complementary data are translated into women’s health digital phenotypes through four layers: data capture, physiological domain mapping, computational phenotyping, and actionable translation. The figure is intended as a conceptual overview; Table 2.

Raw wearable data do not automatically constitute clinical insight. Step counts, skin temperature deviations, heart rate variability, sleep scores, glucose traces, and symptom entries become meaningful only when interpreted within biological, temporal, and clinical contexts. We therefore propose a four-layer framework (Figure 1) for translating wearable signals, together with complementary home-based and patient-generated data, into women’s health digital phenotypes: data capture, physiological domain mapping, computational phenotyping, and actionable translation.

The framework was developed through an iterative, author-led synthesis of the targeted narrative literature, established digital biomarker and validation frameworks, and recurring translational requirements across reproductive life stages. It was refined through multidisciplinary discussion among authors with expertise in artificial intelligence, biomedical informatics, and women’s health. It is a conceptual framework rather than a formal consensus or systematic evidence synthesis. Evidence was interpreted according to study design and intended purpose: validation studies informed performance claims, prospective and interventional studies informed clinical utility, feasibility studies informed implementation, and regulatory guidance informed governance; preprints were used only for emerging methodological developments and framed as research-stage evidence.

### 2.1. Defining Women’s Health Digital Phenotypes

In this perspective, we define a women’s health digital phenotype as a longitudinal, context-aware representation of reproductive, hormone-linked, physiological, behavioral, mental health, or functional status derived from wearable, home-based biochemical, and patient-generated data and interpreted against biologically meaningful and clinically relevant reference points. Throughout this perspective, “women’s health wearables” refers to wearable-centered digital phenotyping systems in which wearable signals remain the primary longitudinal data source but may be complemented by home-based biochemical measurements, patient-reported information, and selected clinical or contextual data. This framing preserves the central role of wearables while recognizing that their interpretation often requires complementary biological and clinical context.

A women’s health digital phenotype should specify five core components: the data modalities used to derive it; the temporal resolution and observation window over which it is computed; the biological or clinical construct it is intended to represent; the uncertainty, confidence, or reliability of the inferred state; and the validation evidence supporting its interpretation in the intended population and use context. It should also be interpretable enough to clarify whether the output represents a measured signal, a derived feature, a predicted state, or an actionable clinical summary. Under this definition, a wearable output that lacks temporal context, uncertainty reporting, biological mapping, or validation evidence should be described more conservatively as a signal, feature, trend, or wellness insight rather than as a digital phenotype.

This definition extends beyond a single digital biomarker. A digital biomarker often refers to an objectively measured, validated digital measure that reflects a biological, physiological, or behavioral process, such as nocturnal HRV, glucose variability, or skin temperature deviation. A digital phenotype, by contrast, integrates multiple signals over time to characterize a state, transition, risk pattern, or response trajectory. In women’s health, clinically meaningful states often emerge from temporal and multimodal patterns rather than isolated measurements. For example, an ovulatory pattern may require temperature, luteinizing hormone (LH), pregnanediol glucuronide (PdG), bleeding, and cycle timing; a postpartum recovery phenotype may combine sleep disruption, activity recovery, HRV, mood symptoms, and lactation context; and a menopause phenotype may link vasomotor events with sleep fragmentation, autonomic changes, fatigue, and next-day functioning. Table 1 summarizes the conceptual progression from raw wearable signals to clinically actionable summaries and provides an operational definition and illustrative women’s health example for each level.

Table 2 translates these distinctions into a signal-to-phenotype taxonomy for women’s health wearable digital phenotyping. The table links common digital signal groups to biological domains, candidate women’s health phenotypes, potential reference standards, and minimum evidence thresholds. This taxonomy clarifies that the same raw signal may support different levels of inference depending on its validation context and intended use.

Importantly, a women’s health digital phenotype does not necessarily require AI. Some clinically useful phenotypes may be derived from descriptive longitudinal summaries, rule-based thresholds, statistical models, or validated scoring systems. In this perspective, we use “AI-enabled” to refer specifically to computational approaches that learn patterns from data, such as machine learning, deep learning, probabilistic sequence models, anomaly detection, clustering, forecasting models, and multimodal fusion methods. AI may add value when wearable and patient-generated data are high-dimensional, temporally dense, nonlinear, incomplete, or highly individualized.

Evidence tiers are cumulative and should be proportional to the intended use and clinical consequence of the claim. Tier 1 establishes technical or analytical validity by confirming that the device or assay accurately captures the intended signal. Tier 2 establishes physiological validity by demonstrating biological plausibility for the inferred construct. Tier 3 establishes clinical validity through association with a meaningful clinical, functional, or experiential state in the intended population. Tier 4 requires evidence of clinical utility, such as improved care, self-management, safety, workflow, or patient-centered outcomes, while Tier 5 addresses implementation and lifecycle readiness, including workflow integration, equity, human oversight, drift monitoring, and post-deployment surveillance. The assignments in Table 2 are therefore fit-for-purpose recommendations rather than universal thresholds. When evidence is inconsistent across tiers, readiness should be determined by the lowest unmet tier, because higher-level clinical associations cannot compensate for inadequate measurement or physiological validity.

**Table 2:** Signal-to-phenotype taxonomy for women’s health wearable digital phenotyping. Evidence tiers: Tier 1 = technical/analytical validity; Tier 2 = physiological validity; Tier 3 = clinical validity; Tier 4 = clinical utility; Tier 5 = implementation and lifecycle readiness. Thresholds are indicative rather than universal and should be adapted to the exact intended use, target population, reference standard, and consequences of error.

Digital Signal Group	Biological Domain	Candidate Women’s Health Digital Phenotype	Potential Reference Standards or Comparators	Minimum Evidence Threshold
Resting heart rate, RMSSD/HRV, nocturnal cardiovascular trends	Autonomic regulation; reproductive physiology; stress physiology	Cycle-related autonomic pattern; pregnancy adaptation trajectory; postpartum recovery strain; menopause-related autonomic instability	ECG-derived HR/HRV; menstrual phase or ovulation confirmed by LH, PdG, serum hormones, or ultrasound where appropriate; validated stress or symptom scales	Tier 1–2 minimum; Tier 3 required for risk prediction; Tier 4–5 if used for monitoring or escalation
Skin temperature, peripheral temperature deviation, basal body temperature, or peripheral temperature-derived estimates	Thermoregulation; ovulatory and luteal physiology; vasomotor physiology	Probable ovulatory thermal shift; nocturnal vasomotor–sleep disruption; deviation from personal thermal baseline	Validated temperature measures; urinary LH/PdG or serum progesterone for ovulation or luteal status; hot flash diaries; skin conductance when relevant; ambient temperature/context data	Tier 1–2 minimum; Tier 3 required for ovulation, fertility, or vasomotor claims; Tier 4–5 for intervention guidance
Sleep duration, sleep timing, sleep efficiency, sleep-stage estimates, restlessness	Sleep and circadian health	Menstrual-cycle sleep vulnerability; pregnancy sleep adaptation; postpartum sleep–recovery mismatch; menopause-related sleep disruption	Polysomnography for sleep-stage claims; actigraphy or validated sleep measures for sleep–wake and duration estimates; validated sleep questionnaires; clinical sleep diagnoses; symptom diaries	Tier 1 required for sleep estimation; Tier 2–3 required for reproductive-stage interpretation; Tier 4–5 if used to guide care
Physical activity, steps, sedentary time, activity recovery	Functional health; recovery; cardiometabolic adaptation	Pregnancy activity adaptation; postpartum functional recovery; fatigue-related activity decline; treatment-response trajectory	Accelerometry validation; patient-reported function; rehabilitation measures; activity logs; clinical recovery assessments	Tier 1–2 minimum; Tier 3 required for recovery or functional phenotypes; Tier 4–5 if used in care pathways
Continuous glucose monitoring metrics, glucose variability, time in range	Metabolic health	Pregnancy hyperglycemia phenotype; gestational diabetes control phenotype; PMOS/metabolic-risk profile; menstrual-cycle metabolic variability; midlife cardiometabolic phenotype	Laboratory glucose; OGTT; HbA1c, where appropriate; clinical diagnosis of gestational diabetes, pre-existing diabetes, pregnancy hyperglycemia, or metabolic disorder; diet and activity context	Tier 1–3 minimum; Tier 4 required for management claims; Tier 5 for remote monitoring or clinical integration
Cuff-based wearable blood pressure, ambulatory blood pressure, pulse-wave features, or cuffless blood pressure estimates	Cardiovascular and vascular risk	Pregnancy hypertensive-risk monitoring; postpartum blood pressure recovery; midlife cardiovascular-risk phenotype	Validated cuff-based blood pressure; ambulatory blood pressure monitoring; clinical diagnosis of hypertensive disorders; pregnancy or postpartum outcomes	Tier 1–3 minimum; Tier 4 required for monitoring or escalation; Tier 5 required for deployed remote-care pathways
Skin conductance, sweating, temperature, HR/HRV, movement, and ambient context during symptom episodes	Thermoregulatory and sympathetic activation	Hot flash or night-sweat phenotype; vasomotor symptom burden; just-in-time intervention target	Hot flash diary; laboratory hot flash monitoring; skin conductance; validated vasomotor symptom scales; ambient temperature and sleep-context data	Tier 2–3 minimum; Tier 4 required for intervention claims; Tier 5 for just-in-time or deployed monitoring systems
Urinary or home hormone measures including LH, E3G, PdG, FSH, estradiol, or progesterone-related measures	Reproductive endocrinology	Hormone-grounded cycle phenotype; ovulation confirmation; luteal function pattern; perimenopause transition profile	Validated urinary assays for biochemical context; serum hormones or ultrasound-confirmed ovulation when clinical confirmation is required; clinical endocrine assessment	Tier 1–2 minimum; Tier 3 required for diagnostic, fertility, or endocrine claims; Tier 4–5 if used for treatment or care decisions
Patient-reported bleeding, pain, mood, vasomotor symptoms, fatigue, sleep quality, work functioning	Symptom burden; mental health; functional status	Premenstrual symptom phenotype; postpartum mood-risk phenotype; menopause functional-burden phenotype; treatment-response profile	Validated symptom scales; structured diaries; screening tools; diagnostic interviews where relevant; clinical assessment for high-risk mental health or functional outcomes	Tier 3 minimum for clinical interpretation; Tier 4 required for referral, treatment, or monitoring claims; Tier 5 for integration into care workflows
Fetal heart rate, uterine activity, maternal–fetal monitoring signals	Maternal–fetal physiology	High-risk pregnancy monitoring phenotype; fetal or uterine activity monitoring profile	Clinical fetal monitoring standards; ultrasound or obstetric assessment; maternal–fetal outcomes; triage and safety endpoints	Tier 1–3 minimum; Tier 4 required for clinical use; Tier 5 required for any deployed monitoring, escalation, or safety-critical pathway

### 2.2. Four-Layer Framework for Women’s Health Digital Phenotyping

#### 2.2.1. Layer 1: Data Capture

The first layer is the acquisition of longitudinal, time-stamped data from wearable and patient-reported sources. These data include passive physiological and behavioral signals such as heart rate, HRV, skin temperature, respiratory patterns, sleep, activity, and glucose. They may be enriched by biochemical context from home hormone testing, including LH, E3G, PdG, FSH, estradiol, or progesterone-related measures, and by patient-reported information on bleeding, pain, vasomotor symptoms, mood, fatigue, sleep quality, medications, sexual activity, and functional impact. The key requirement at this layer is not simply device availability, but signal validity and metadata quality. Multimodal phenotyping also requires harmonization across devices, platforms, and data sources, because similar measures may be collected using different sensors, sampling rates, preprocessing pipelines, proprietary algorithms, and sensing assumptions. In addition, sampling frequency, wear time, device placement, firmware version, missingness, motion artifact, skin tone, body habitus, environmental conditions, and adherence all influence interpretation. For example, skin temperature from a ring should not be interpreted as core body temperature or direct progesterone activity, and wearable-derived sleep duration is not equivalent to polysomnography-derived sleep architecture.

#### 2.2.2. Layer 2: Physiological Domain Mapping

The second layer organizes heterogeneous signals into biologically meaningful domains relevant to women’s health. These include autonomic function, sleep and circadian health, thermoregulation, metabolism and glucose regulation, reproductive endocrinology, mental health and stress, and functional health. This layer is where biological plausibility is established. For example, wearable-derived resting heart rate and root mean square of successive differences (RMSSD) have been shown to fluctuate across the menstrual cycle, supporting the relevance of longitudinal cardiovascular signals as non-invasive markers of reproductive physiology [2]. However, physiological mapping also requires caution. Temperature, HRV, sleep disruption, glucose variability, and sweating are not specific to one reproductive state. The same signal may reflect ovulation, pregnancy adaptation, infection, medication effects, stress, environmental heat, vasomotor symptoms, or sleep disruption. Therefore, physiological proxies should not be described as direct hormone measurements unless biochemical data are collected.

#### 2.2.3. Layer 3: Computational Phenotyping

The third layer transforms longitudinal data into interpretable patterns, predictions, and risk profiles. Because reproductive and hormonal physiology varies substantially between women, computational phenotyping should prioritize within-person change and personalized baselines. Relevant tasks include anomaly detection, short-term forecasting, risk stratification, symptom clustering, state and transition detection, and multimodal fusion. These methods may support the identification of cycle transitions, probable ovulation, pregnancy adaptation, postpartum recovery patterns, vasomotor symptom burden, treatment response, and functional decline.

Computational phenotyping may use descriptive analytics, rule-based logic, statistical modeling, machine learning, deep learning, or other AI-enabled approaches. AI is not required for every task; validated thresholds or longitudinal trend summaries may be sufficient for personal awareness or routine monitoring. Descriptive and clinically interpretable tasks may also be addressed using generalized additive models, mixed-effects models, or tree-based models to characterize personal baselines, within-person change, and explainable trends. More complex AI approaches may be useful for multimodal fusion, individualized baseline learning, anomaly detection, forecasting, symptom clustering, and risk stratification across heterogeneous longitudinal data streams.

For sequence-aware applications, such as ovulation-window estimation, symptom-flare forecasting, postpartum recovery trajectories, or vasomotor–sleep disruption patterns, recurrent neural networks, temporal convolutional networks, transformers, state-space models, hidden Markov models, and temporal fusion models may better capture time dependence, irregular sampling, and transitions between states. Recent wearable foundation models and sensor–language models, such as SensorLM, further demonstrate the potential of reusable representations for multimodal wearable data; however, these remain research-stage approaches and require women’s health-specific validation before clinical use [3,4]. No single approach is inherently optimal. Model selection should reflect the intended use, temporal structure and quality of the data, missingness, sample size, available reference labels, interpretability and uncertainty requirements, validation evidence, and the consequences of false-positive or false-negative outputs.

#### 2.2.4. Layer 4: Actionable Translation

The fourth layer converts digital phenotypes into outputs that are understandable, proportionate, and useful. In this framework, an actionable phenotype is a sufficiently validated and interpretable phenotype that is linked to a predefined response, such as reassurance, self-management, routine discussion, clinician review, or urgent escalation, with the intended user, uncertainty, decision threshold, and responsible actor clearly specified. Clinical utility refers to prospectively demonstrated improvement in prespecified patient-, clinician-, or health-system outcomes, such as earlier appropriate referral, improved symptom management, reduced unnecessary visits or alerts, greater workflow efficiency, or improved patient experience, without unacceptable harm or burden.

For patients, actionable translation should support sensemaking by helping users interpret wearable outputs alongside symptoms, lived experience, and varying levels of menstrual and reproductive health literacy. Outputs should use plain language, communicate uncertainty, and avoid implying that algorithmic signals override women’s own experiences. For clinicians, it may include concise pre-visit summaries for fertility, obstetric, postpartum, primary care, or menopause consultations. For remote monitoring, it may include risk stratification, treatment-response tracking, and referral prioritization. Actionable outputs could include flagging persistent nocturnal vasomotor–sleep disruption for treatment review, identifying poor postpartum recovery trajectories for earlier follow-up, or summarizing glucose–activity patterns to guide gestational diabetes counseling. The goal is not to generate more dashboards or alerts, but to deliver fewer, better signals that support self-management, clinical judgment, shared decision-making, and timely escalation when needed.

#### 2.2.5. Operationalizing the Four-Layer Framework

To facilitate practical application, Table 3 translates the four-layer framework into operational requirements for researchers, developers, and health-technology stakeholders. It outlines representative minimum inputs, key analytical and decision points, reportable outputs, validation criteria, and illustrative clinical use cases for each layer, while recognizing that requirements should be adapted to the intended population, use context, and clinical consequence.

#### 2.2.6. Illustrative Workflow: Postpartum Recovery Phenotyping

As an illustrative workflow, consider longitudinal postpartum recovery monitoring. At Layer 1, a wearable-centered system could integrate resting heart rate, HRV, sleep fragmentation, activity and mobility recovery, together with patient-reported mood, pain, fatigue, bleeding, lactation demands, and perceived functioning. At Layer 2, these inputs would be mapped to autonomic regulation, sleep and circadian health, functional recovery, mental health, and symptom burden, while accounting for postpartum week, delivery mode, complications, medication use, and caregiving context. At Layer 3, a personalized longitudinal model could compare the evolving pattern with the individual’s antepartum baseline, early postpartum trajectory, and expected recovery range, integrating data completeness and uncertainty to identify a persistent recovery-mismatch phenotype characterized by sustained physiological strain, limited sleep recovery, reduced activity, and worsening symptoms rather than a single abnormal measurement. At Layer 4, the phenotype could be translated into a concise, tiered output: reassurance and self-management support when recovery remains within the expected range; routine postpartum discussion when deviations persist; or earlier clinician review when multimodal deterioration or concerning symptoms emerge. The clinician-facing summary could display the observation window, deviation from personal baseline, contributing signals, confidence level, and recommended response, thereby converting continuous data into a clinically interpretable trajectory rather than an additional stream of alerts.

### 2.3. Validation as the Bridge from Measurement to Meaning

Across all four layers, validation is the bridge between measurement and meaning. The V3 framework emphasizes verification, analytical validation, and clinical validation as core requirements for determining whether biometric monitoring technologies are fit for purpose [5]. Applied to women’s health wearables, this means asking three sequential questions: Does the device accurately capture the signal? Does the algorithm correctly derive the intended measure? Does that measure meaningfully represent a relevant biological, clinical, functional, or experiential state in the intended population?

This validation chain must be context-specific. Where relevant, wearable-derived proxies should be evaluated alongside biochemical or molecular measures. Emerging placental extracellular-vesicle profiles provide one research-stage example of complementary biological context in pregnancy [6]. The meaning of reduced HRV, increased nocturnal temperature, sleep fragmentation, or activity decline nevertheless remains dependent on reproductive stage and clinical context. The translational value of women’s health wearables will therefore depend not on the number of signals collected, but on whether those signals can be linked to reproductive biology, interpreted through validated and transparent models, and translated into actions that are meaningful to women and useful to clinicians.

### 2.4. Positioning the Proposed Framework Relative to Existing Digital Biomarker Frameworks

Our framework is intended to complement, rather than replace, existing digital biomarker frameworks and pipelines. The V3 framework provides a general fit-for-purpose validation structure for biometric monitoring technologies by emphasizing verification, analytical validation, and clinical validation [5]. The Digital Biomarker Discovery Pipeline (DBDP) extends this field by providing an open-source software ecosystem for digital biomarker development using mHealth and wearable data, with emphasis on transparency, reusable workflows, preprocessing, feature extraction, machine learning, and end-to-end digital biomarker discovery [7]. More broadly, digital biomarker development pipelines focus on transforming raw sensor data into validated digital measures that can support health-related inference.

The framework proposed here differs in scope and intent. Rather than focusing primarily on generic biometric validation or computational pipeline development, it provides a women’s health-specific translational structure for interpreting wearable-centered signals across reproductive life stages. Its unique contribution is the explicit integration of reproductive biology, hormone-linked interpretation, life-stage context, multimodal patient-generated data, proportional evidence thresholds, and governance for sensitive reproductive and hormonal data. This distinction is important because the same wearable signal may have different meanings across menstruation, fertility, pregnancy, postpartum recovery, perimenopause, menopause, lactation, hormone therapy exposure, and symptom burden. Therefore, our framework positions digital phenotyping not only as a technical or analytic process but also as a biologically contextualized and clinically proportionate process that links data capture to meaningful action across women’s healthcare.

## 3. Wearable Digital Phenotyping Across the Reproductive Lifespan

**Figure 2:**
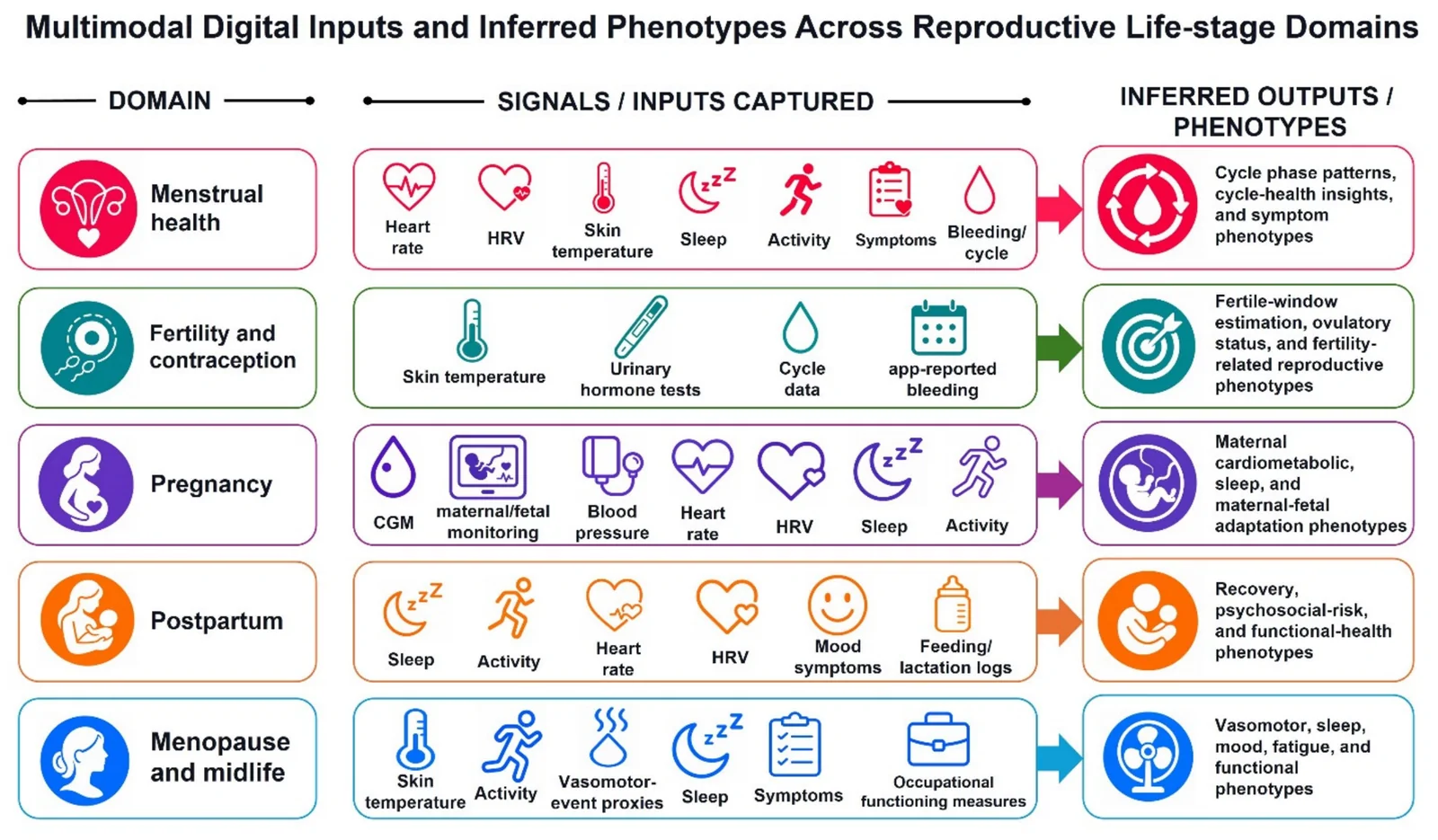
Multimodal digital inputs and inferred phenotypes across reproductive life-stage domains. The figure maps key women’s health domains to representative wearable-derived and patient-reported inputs and the inferred phenotypes they may support.

Women’s health wearables are most clinically meaningful when interpreted within specific reproductive and life-stage contexts rather than treated as a single technology category. Across menstruation, fertility, pregnancy, postpartum recovery, and menopause, wearable-derived signals—often complemented by connected biosensors, home-based testing, and self-reported symptoms—can help document longitudinal physiology, estimate reproductive events, detect deviations from the personal baseline, support self-management and remote monitoring, and inform clinical decision-making. However, the required level of evidence should increase as the intended use becomes more clinically consequential. A cycle-awareness insight, fertility estimate, pregnancy-monitoring alert, postpartum depression risk signal, and menopause treatment-response profile each require different levels of validation, governance, and clinical integration. Whereas Table 2 provides an operational signal-to-phenotype taxonomy, Figure 2 highlights life-stage application mapping and illustrates how the composition and interpretation of wearable-centered digital phenotyping ecosystems differ across reproductive stages.

### 3.1. Menstrual Health and Fertility

Menstrual health and fertility are among the most active areas of innovation in women’s health wearables, although their level of clinical maturity varies substantially by intended use and validation standard [8]. Wearables can capture signals that vary across the menstrual cycle, including resting heart rate, HRV, skin temperature, estimated respiratory rate, sleep, and physical activity, as well as bleeding patterns, symptoms, and urinary hormones when linked to companion tools. Together, these signals offer an opportunity to move beyond calendar-based tracking toward more physiologically informed representations of menstrual function. Large wearable studies have shown systematic within-person fluctuations in cardiovascular parameters across the menstrual cycle, supporting the potential of resting heart rate and HRV as non-invasive markers of reproductive physiology [2]. Temperature monitoring is also relevant because post-ovulatory progesterone is associated with a thermogenic shift, although peripheral skin temperature should be treated as a proxy rather than a direct measure of core temperature or progesterone activity [9].

The main promise in this domain is the development of hormone-aware digital phenotypes that can support cycle literacy, ovulation estimation, irregular-cycle detection, symptom-risk profiling, and research into conditions such as polyendocrine metabolic ovarian syndrome (PMOS; formerly polycystic ovary syndrome [PCOS]), premenstrual disorders, postpartum cycle return, and perimenopause. PMOS is the consensus name introduced in 2026 to better reflect the condition’s endocrine, metabolic, reproductive, and broader health features [10]. However, the major limitation remains endocrine validation. Wearable-derived phase or ovulation estimates are often evaluated against heterogeneous comparators, including urinary LH, PdG, multi-hormone testing, ultrasound, serum hormones, or incompletely specified standards, which limits comparability across studies [11].

Fertility-oriented applications require additional caution because their outputs may influence conception planning or contraceptive behavior. For example, a study using Oura Ring data reported promising performance against ovulation prediction kits as the comparator, but urinary LH testing, wearable-derived temperature shifts, serum progesterone confirmation, and ultrasound-confirmed ovulation are not interchangeable reference standards [12]. The responsible path forward is to clearly distinguish cycle awareness, ovulation prediction, ovulation confirmation, fertile-window estimation, contraception, and endocrine diagnosis. Cycle awareness is primarily descriptive, whereas ovulation prediction and fertile-window estimation are probabilistic outputs. Ovulation confirmation requires biochemical or ultrasound-based evidence, contraception support requires prospective effectiveness and failure-rate data, and endocrine diagnosis requires validated clinical and laboratory criteria with appropriate clinician oversight. Datasets such as mcPHASES, which link wearable physiology, CGM, self-reported symptoms, and urinary hormones including LH, E3G, and PdG, provide an important model for developing hormone-grounded wearable algorithms rather than relying on calendar assumptions alone [13,14].

### 3.2. Pregnancy and Postpartum

Pregnancy and postpartum recovery are high-value contexts for wearable digital phenotyping because they involve rapid physiological, behavioral, metabolic, and psychosocial changes that are often incompletely captured during routine care. In pregnancy, wearable-derived heart rate, HRV, sleep, temperature, activity, blood pressure, glucose, and fetal-monitoring signals may help characterize maternal adaptation over time. Studies using wearable data have described gestational trajectories in cardiovascular, cardiorespiratory, sleep, activity, and temperature metrics, suggesting that the greatest value may lie in detecting deviations from expected individual or gestational patterns rather than interpreting isolated measurements [15,16]. Among wearable and sensor-based technologies used in pregnancy, continuous glucose monitoring has one of the clearest clinical measurement targets because it captures interstitial glucose trajectories relevant to gestational diabetes, including nocturnal glucose, postprandial excursions, glycemic variability, and time in range [17,18].

The main promise of pregnancy wearables is earlier recognition of clinically relevant trajectories, improved self-management, and remote monitoring for selected high-risk contexts. However, pregnancy applications require safety-focused validation because errors may affect both mother and fetus. Blood pressure risk cannot be inferred reliably from heart rate or HRV alone, and fetal or uterine monitoring technologies require strong evidence for signal quality, clinical interpretation, triage value, and outcome benefit. Remote maternal–fetal monitoring and wearable fetal ECG systems are promising, but larger studies are needed to establish safety, escalation pathways, and clinical utility [19,20]. Therefore, AI-derived prediction, risk-stratification, and clinical decision-support outputs in pregnancy and postpartum care should be interpreted according to their validation stage and intended use: exploratory models may support research or hypothesis generation, clinically validated models may support adjunctive monitoring, and only prospectively evaluated tools with demonstrated clinical utility and defined escalation pathways should inform care decisions.

Postpartum applications remain comparatively underdeveloped despite major changes in sleep, activity, lactation, pain, bleeding, mood, autonomic regulation, and caregiving burden. The American College of Obstetricians and Gynecologists (ACOG) emphasizes that postpartum care should be an ongoing process rather than a single encounter, and wearable technologies align naturally with this model by capturing physiological and behavioral changes between clinical visits [21]. Wearable-derived sleep fragmentation, activity recovery, resting heart rate, HRV, and device engagement may provide useful context for maternal recovery and mood vulnerability [22]. Early studies using All of Us wearable data suggest that consumer-derived features may help identify patterns associated with postpartum mood symptoms, although the evidence remains exploratory and these tools should support screening, discussion, and referral rather than independent risk prediction or diagnosis [23,24,25,26]. Postpartum wearable models should be designed to recognize a recovery-mismatch pattern, defined by persistent sleep loss, physiologic strain, reduced activity recovery, lactation demands, and mood symptoms that persist beyond expected postpartum adaptation and warrant clinical review [27,28]. Postpartum phenotyping should also distinguish early, intermediate, and late recovery phases and incorporate delivery-specific outcomes such as wound or perineal healing, pelvic-floor and urinary symptoms, return to mobility or work, and persistence of hypertensive or metabolic complications. Models should also separate maternal physiological change from infant-driven sleep disruption and caregiving-related non-wear, which may otherwise mimic deterioration.

### 3.3. Menopause and Midlife Health

Perimenopause and menopause represent especially important frontiers for longitudinal digital phenotyping, including AI-enabled approaches when the goal is to detect episodic, multidimensional, and individualized symptom patterns. The menopause transition can include irregular bleeding, vasomotor symptoms, sleep disturbance, mood changes, fatigue, cognitive complaints, cardiometabolic changes, and reduced work functioning. These symptoms fluctuate over months to years and differ substantially between women [29]. Wearables could therefore help characterize menopause as a dynamic profile of physiology, symptoms, behavior, and function rather than relying solely on retrospective symptom recall.

Vasomotor symptoms are a particularly promising target because hot flashes and night sweats may be reflected in thermoregulation, sweating, skin conductance, heart rate, HRV, movement, and sleep disruption. Recent work on hot flash prediction suggests the possibility of just-in-time interventions before symptoms are consciously perceived [30]. However, hot flash detection is physiologically complex because sweating and temperature changes may also reflect ambient heat, exertion, anxiety, alcohol, illness, medications, or sleep stage. Multi-sensor models integrating skin conductance, skin temperature, HR/HRV, movement, sleep context, and self-report are likely to be more robust than single-signal approaches.

Beyond vasomotor symptoms, menopause phenotyping should integrate genitourinary symptoms, mood and cognitive complaints, cardiometabolic changes, sexual health, and daily or work functioning, while accounting for aging, comorbidities, and hormonal or non-hormonal therapies. Longitudinal studies should determine whether these profiles improve symptom stratification and treatment-response monitoring against validated clinical and patient-reported endpoints.

The clinical promise of menopause wearables lies in capturing how episodic vasomotor events interact with sleep fragmentation, autonomic instability, mood vulnerability, fatigue, and next-day functioning. Rather than counting hot flashes alone, wearable models could identify clinically meaningful phenotypes such as nocturnal vasomotor–sleep disruption, stress-reactive symptom flares, recovery failure after poor sleep, or activity decline linked to fatigue and thermoregulatory burden. These patterns may generate hypotheses for more personalized symptom management and future treatment-response studies involving hormone therapy, non-hormonal vasomotor treatments, sleep-focused interventions, behavioral support, or workplace accommodations. Early studies of cooling devices and digital therapeutic programs suggest that technology-enabled approaches may reduce vasomotor symptom burden and improve related sleep or behavioral health outcomes [31,32]. Thus, menopause digital phenotyping should move beyond symptom counting toward actionable monitoring that identifies high-burden phenotypes, guides behavioral and therapeutic adjustments, and tracks response across vasomotor symptoms, sleep, mood, fatigue, and daily functioning.

## 4. Evidence and Implementation Readiness

**Figure 3:**
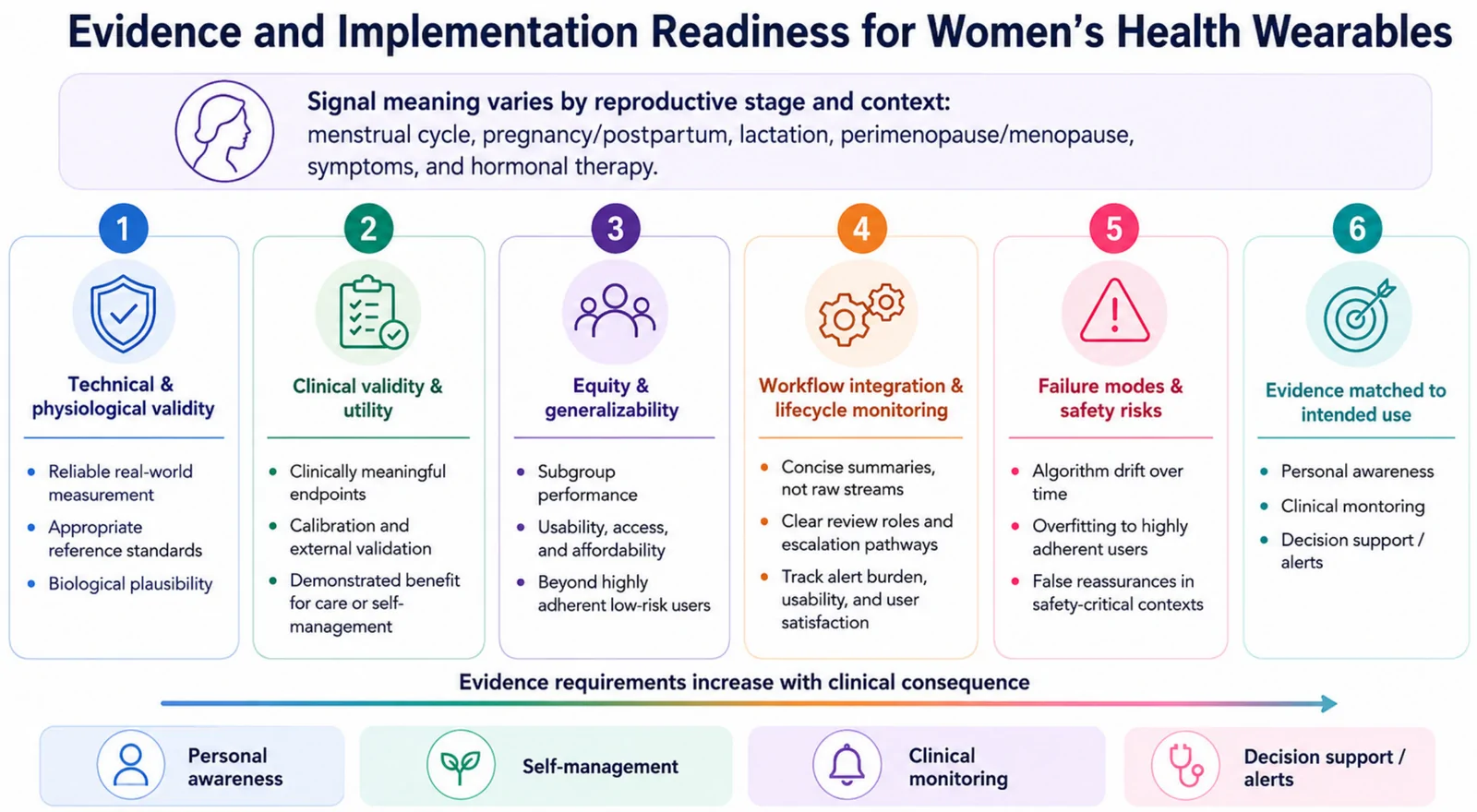
Evidence and Implementation Readiness for Women’s Health Wearables.

Women’s health wearables should not be evaluated by sensor accuracy alone. A device may accurately measure heart rate, skin temperature, sleep duration, glucose, activity, or movement, yet still fail to provide clinically meaningful insight if the signal is not biologically interpretable, clinically relevant, or usable within care. The central question is whether the measured signal is fit for purpose in the intended population, reproductive stage, clinical context, and decision pathway. Building on the V3 framework [5], women’s health applications additionally require physiological validity, clinical utility, equity, workflow integration, and lifecycle monitoring. Figure 3 summarizes this framework and illustrates how validation requirements, equity considerations, workflow integration, and lifecycle monitoring should be matched to intended clinical use.

### 4.1. Technical and Physiological Validity

Technical validity asks whether the device accurately captures the intended signal under real-world conditions. In women’s health, this must be assessed across physiological states that may alter sensor performance, including pregnancy-related edema, postpartum sleep disruption, altered skin perfusion, night sweats, body-composition changes, hormone therapy use, and vasomotor symptoms. Validation should address sensor precision, calibration, sampling rate, motion artifact, device placement, firmware version, wear time, missingness, and appropriate reference standards, such as ECG for heart rate and HRV, polysomnography for sleep, validated CGM or laboratory comparators for glucose, validated temperature measures, and clinical standards for fetal heart rate or uterine activity monitoring. The EVIDENCE checklist emphasizes transparent reporting of the device, algorithm, data collection conditions, and validation approach [33]. For women’s health studies, this transparency should also include reproductive stage, pregnancy or postpartum status, menopause status, BMI, skin tone, device fit, and symptom context [34].

Physiological validity asks whether the captured signal reflects the biological process of interest. Temperature rise may correlate with luteal-phase physiology but should not be described as a direct progesterone measurement. Reduced HRV may reflect stress, poor sleep, illness, medication exposure, pregnancy adaptation, or hormonal transition. Skin conductance may support hot flash detection, but sweating may also reflect heat, exertion, anxiety, infection, medication effects, or sleep stage. Physiological validity therefore requires biologically plausible models and comparison with appropriate standards, including hormone assays, symptom diaries, clinical events, diagnostic tests, or established physiological markers. Without this step, wearable outputs may be technically accurate but clinically ambiguous.

### 4.2. Clinical Validity and Clinical Utility

Clinical validity asks whether a wearable-derived feature detects, estimates, or predicts a clinically meaningful state. The International Medical Device Regulators Forum guidance on Software as a Medical Device emphasizes valid clinical association, analytical validation, and clinical performance as core components of clinical evaluation [35]. In women’s health, clinical validity should be tied to clearly defined endpoints, such as ovulation confirmation, cycle irregularity, gestational diabetes control, preeclampsia risk, preterm birth risk, postpartum depression risk, hot flash burden, sleep disruption, treatment response, or functional impairment. Clinical validity should not be inferred from correlation alone. Quantitative readiness criteria should be prespecified according to the intended use and clinical consequence. Relevant measures may include sensitivity, specificity, predictive values, agreement, discrimination, calibration, confidence intervals, external validation, and subgroup performance. Acceptable thresholds should reflect the clinical risk and care pathway rather than universal cutoffs. External validation should be conducted in an independent cohort that differs meaningfully in clinical site, population characteristics, and, where relevant, device or software version, with subgroup performance and calibration reported separately. Digital biomarker development similarly requires clear reference measures, intended use, and interpretation pathways before sensor-derived measures can function as health-relevant biomarkers [36].

Clinical utility is the next and often missing step. A wearable may estimate a fertile window, classify sleep disruption, flag glucose variability, or quantify vasomotor symptom burden, but its value depends on whether it improves care, self-management, patient experience, shared decision-making, or clinical efficiency. Clinical utility requires pragmatic studies, care-pathway evaluations, and patient-centered outcomes. In pregnancy, utility may mean fewer unnecessary visits without missed complications. In postpartum care, it may mean earlier screening and referral for mood or recovery concerns. In menopause, it may mean more personalized symptom management or treatment-response monitoring. The challenge is to move from measuring endpoints to demonstrating meaningful benefit [37].

### 4.3. Equity and Generalizability

Equity and generalizability ask whether the wearable works across the populations it is intended to serve. Women’s health technologies should be evaluated across age, ethnicity, skin tone, BMI, pregnancy status, postpartum status, cycle regularity, hormone therapy exposure, perimenopause, disability, geography, language, and socioeconomic context. A model trained mainly on young, healthy, highly adherent users with regular cycles may fail in women with PMOS, irregular cycles, darker skin tones, obesity, shift work, chronic disease, postpartum physiology, or menopausal transition. Equity should therefore be assessed across sensor performance, algorithmic performance, adherence, comprehension, affordability, language access, and clinical access.

Human factors are central to generalizability because real-world performance depends not only on measurement accuracy but also on whether users can engage with the tool, interpret outputs appropriately, and act on recommendations without excessive burden [38]. Digital pregnancy care studies illustrate this point: engagement can vary substantially across self-monitoring tasks, reinforcing that even clinically relevant tools must be designed around motivation, effort, health literacy, and daily-life constraints [39]. For women’s health wearables, equity should therefore be treated as a deployment property, not a post hoc subgroup analysis: a tool is not generalizable unless it remains usable, interpretable, affordable, and clinically actionable for the women most likely to experience fragmented care, atypical physiology, or barriers to digital health access.

Deployment in low- and middle-income countries presents additional challenges related to device and subscription costs, smartphone and internet access, digital literacy, language localization, data infrastructure, continuity of sensor supplies, and limited capacity for clinical review or referral. Wearable programs should therefore be designed for local care pathways rather than assuming continuous connectivity, specialist availability, or integration with advanced electronic health records. At the same time, lower-cost sensors, offline or low-bandwidth functionality, community health-worker-supported models, and integration with existing maternal and primary care services may create opportunities to extend longitudinal monitoring where access to facility-based care is limited. Evaluation in these settings should include affordability, usability, infrastructure requirements, data governance, and whether identified risks can be linked to an accessible and timely clinical response.

### 4.4. Workflow Integration and Lifecycle Monitoring

Implementation readiness requires wearable-derived insights to be integrated into care without increasing burden or causing harm. Raw streams of heart rate, sleep, temperature, glucose, activity, symptoms, or hormone-linked data should therefore be converted into concise, interpretable summaries. Although clinicians recognize the value of consumer wearables and patient-generated data, concerns remain regarding workload, interpretation, patient expectations, access, privacy, and education [40]. Summaries should report the observation window, data completeness, comparison with personal baseline, relevant deviations, uncertainty, and an appropriate response level. Patient-facing outputs should support self-management without implying diagnostic certainty, whereas clinician-facing reports should reduce recall burden and facilitate targeted discussion. User-centered research similarly indicates that patients value personalized education and emotional support, while clinicians prioritize evidence of utility, workflow integration, and concise actionable summaries [41].

Practical adoption also depends on regulatory, financial, and organizational readiness. Systems that make diagnostic, monitoring, or treatment claims may require evaluation under applicable medical-device or Software as a Medical Device pathways. Reimbursement and health-system adoption will depend on evidence of clinical utility, cost-effectiveness, defined clinical responsibilities, interoperability, and sustainable staffing. Scalability further requires standardized data integration, manageable review and alert workflows, support across devices and populations, and implementation models that do not transfer excessive cost or workload to patients or clinicians.

Risk stratification and remote monitoring require calibrated, externally validated outputs linked to predefined actions. Although early-warning systems may support earlier detection of deterioration, inadequate specificity can generate substantial alert burden [42]. Women’s health outputs should therefore be tiered into personal insight, self-management guidance, routine discussion, clinician review, and urgent escalation, because isolated changes in cycle patterns, sleep, vasomotor symptoms, glucose, or mood are not necessarily pathological. Safety and usability evaluations should assess false-positive and false-negative performance, interpretability, alarm burden, workload, and user satisfaction [43,44].

In pregnancy pathways, safety evaluation should explicitly quantify false-negative risks for clinically important maternal or fetal deterioration, false-positive alert burden, and performance against prespecified maternal–fetal endpoints. Escalation thresholds, reviewer roles, after-hours coverage, documentation requirements, and final clinical accountability should be defined before deployment, while preserving symptom-based access to care regardless of the wearable output.

Lifecycle monitoring should focus on whether performance and clinical manageability are maintained after deployment. Relevant measures include data completeness, patient engagement, review timeliness, escalation outcomes, performance drift, safety incidents, and unintended consequences. Health systems should also document device and software updates, model versions, and changes in operational responsibilities [45,46]. This ongoing oversight is particularly important when wearable outputs influence reassurance, self-management, clinical review, or escalation across sensitive reproductive and life-stage contexts.

### 4.5. Failure Modes and Safety Risks

Women’s health wearable systems may fail even when individual sensors appear technically accurate. One important failure mode is algorithm drift, which may occur as device firmware, sensor hardware, user behavior, population characteristics, clinical practice, and reproductive-stage distributions change over time. In women’s health, drift is especially relevant because the meaning of wearable signals may shift across life stages, illness, medication use, and environmental context. A model trained on one device version, life stage, or user population may therefore become less reliable when applied to another. Drift monitoring, periodic recalibration, post-deployment performance surveillance, and predefined procedures for model updates are essential, particularly for AI-enabled systems used beyond personal awareness. More broadly, findings across wearable phenotyping studies are not always consistent or independently replicated. Apparent associations and model performance may vary according to device generation, sensor placement, preprocessing methods, reference standards, cohort composition, reproductive stage, and adherence, and performance observed in retrospective or highly selected cohorts may therefore attenuate during prospective real-world deployment.

A second failure mode is overfitting to high-adherence users. Wearable datasets often overrepresent individuals who can afford devices, tolerate continuous monitoring, and consistently wear or synchronize devices, while underrepresenting women with fragmented care, lower digital access, chronic disease, disability, shift work, language barriers, or lower adherence. As a result, models may appear accurate in development cohorts but perform poorly in the populations most likely to benefit from earlier monitoring or support. A third and particularly important failure mode is false reassurance, especially in pregnancy, postpartum recovery, and other safety-critical contexts. For example, a normal wearable-derived summary should not be interpreted as excluding preeclampsia, fetal compromise, infection, hemorrhage, or other clinically important complications unless the tool has been validated for that specific intended use. Wearable outputs should therefore communicate uncertainty, define what the tool can and cannot detect, preserve symptom-based escalation, and remain embedded within clinician-governed care pathways.

### 4.6. Matching Evidence Thresholds to Intended Use

Evidence requirements should be proportional to the clinical consequences of the intended use. Accordingly, exploratory or retrospectively associated wearable signals should not be described as clinically actionable, while claims of clinical deployment should be reserved for tools supported by Tier 4–5 evidence and prospective evaluation in the intended population and care pathway. Furthermore, evidence thresholds cannot be generalized by application category alone; they should be determined by the exact claim, target population, role of the output in the care pathway, quality of the reference standard, and consequences of false-positive or false-negative results. Tools used for personal awareness may require technical validity and physiological plausibility, whereas tools used for contraception, pregnancy monitoring, postpartum depression risk, vasomotor symptom management, or clinical decision support require stronger clinical validation, utility evidence, equity assessment, workflow integration, and lifecycle safeguards. This proportional approach helps distinguish consumer wellness insights from clinical-grade digital biomarkers and medical-device-level claims.

## 5. Governance, Equity, and Trust in Women’s Health Wearables

Women’s health wearables generate data that may reveal menstrual patterns, fertility intentions, pregnancy status, contraception use, pregnancy loss history, lactation patterns, menopausal symptoms, mental health status, sleep behavior, activity routines, location-linked habits, and workplace functioning. These data should not be treated as generic wellness information because they may disclose highly sensitive reproductive, behavioral, and social information. Responsible women’s health digital phenotyping should therefore treat governance, equity, and trust as core design requirements rather than downstream compliance tasks.

### 5.1. Reproductive Data Are Not Generic Wellness Data

Reproductive and hormonal data require stronger safeguards than generic activity or wellness metrics. A wearable-derived signal may appear clinically neutral, such as sleep disruption, temperature change, or reduced activity, but its interpretation may imply ovulation, pregnancy, miscarriage, postpartum recovery, menopausal symptoms, or mental health vulnerability. This creates a higher duty of care for how data are collected, interpreted, shared, and communicated. Users should understand whether an output represents a measured signal, a derived feature, or an algorithmic inference. Clinicians should also be able to distinguish between direct measurements, physiological proxies, and predictive model outputs. Without this transparency, women may be exposed to misleading claims, inappropriate reassurance, unnecessary anxiety, or unintended disclosure of sensitive reproductive information.

Concrete risks may arise when reproductive inferences are used outside the context anticipated by the user. For example, an employer-sponsored wellness program could expose menstrual, pregnancy, or menopause-related patterns to workplace decision-making; an insurer could use inferred health or pregnancy risk for profiling; and fertility or pregnancy signals could be used for targeted advertising without explicit consent. Wearable data may also permit pregnancy or miscarriage-related inference from temperature, activity, sleep, or symptom patterns, even when users have not directly disclosed these events. The potential consequences are especially context-dependent, where reproductive health information may carry legal, occupational, insurance, social, or geopolitical risks.

### 5.2. Consent, Secondary Use, and User Control

Consent for women’s health wearables should be treated as an ongoing governance process rather than a one-time acceptance of a privacy policy. Because these technologies may collect reproductive, hormonal, behavioral, and mental health-related data, users should be able to understand and control how their data are collected, stored, analyzed, shared, and reused. Meaningful consent should be granular, revocable, and specific to the intended purpose, including personal feedback, clinical sharing, research, product improvement, advertising, third-party analytics, and AI model training. Studies of menstrual tracking and FemTech apps show that users are particularly concerned about privacy, accuracy, data security, and whether their data are used in ways that feel purposeful, transparent, and respectful [47,48]. The Flo Health case further illustrates the importance of enforceable governance when sensitive health information is shared with external analytics providers despite privacy assurances [49]. For women’s health wearables, secondary data use should therefore be opt-in, purpose-limited, auditable, and separated from core device functionality whenever feasible. Users should also have clear mechanisms to access, export, delete, and withdraw consent for future non-essential uses.

### 5.3. Bias, Access, and Responsible Deployment

Responsible deployment should address affordability, accessibility, differential sensor performance, and the risk that models developed in young, healthy, highly adherent users may widen existing disparities. Governance plans should therefore prespecify subgroup evaluation across relevant reproductive, physiological, and device-use characteristics, including reproductive stage, skin tone, BMI, pregnancy or postpartum status, menopause status, and device adherence. Where sample sizes are insufficient, uncertainty and limitations should be reported rather than the subgroup assessment being omitted.

The NIST AI Risk Management Framework emphasizes governance, risk mapping, measurement, and management throughout the AI lifecycle [50]. Applied to women’s health wearables, this requires bias assessment, drift monitoring, human oversight, incident reporting, and corrective action to be embedded in design, validation, and deployment. Implementation planning should also consider device and subscription costs, connectivity, language, digital literacy, and access to clinical support. Responsible deployment should ensure that wearable innovation does not primarily benefit already advantaged users while widening disparities among women with greater clinical and digital health needs.

## 6. Research Priorities for the Next Phase of Women’s Health Wearables

The next phase of women’s health wearables should move from device-centered innovation toward biologically grounded, clinically validated, equitable, and implementation-ready digital phenotyping. Six priorities can guide this transition.

First, the field needs hormone-grounded longitudinal cohorts. Many current studies infer reproductive or hormonal states from temperature, heart rate, HRV, sleep, or activity, but relatively few incorporate repeated biochemical measurements. Future cohorts should integrate wearable physiology with point-of-care diagnostics, digital biosensors, and urinary or serum biomarkers, including LH, E3G, PdG, estradiol, progesterone, FSH, glucose, inflammatory markers, and other clinically relevant laboratory measures. Datasets such as mcPHASES provide an important early model by linking wearable physiology, CGM, self-reported symptoms, and urinary hormones across menstrual cycles [13,14]. Larger and more diverse cohorts should extend this approach to irregular cycles, PMOS, postpartum cycle return, perimenopause, hormone therapy exposure, and reproductive endocrine disorders.

Second, research should expand beyond fertility and pregnancy to postpartum and menopause. These transitions remain comparatively underdeveloped despite substantial unmet clinical need. Postpartum recovery involves sleep disruption, lactation, mood vulnerability, autonomic recalibration, activity recovery, and physical healing. Perimenopause and menopause involve fluctuating vasomotor, sleep, mood, fatigue, metabolic, and functional symptoms over months to years. Longitudinal monitoring is therefore particularly suited to these under-measured life stages [1].

Third, safety-critical applications require prospective validation. Pregnancy monitoring, gestational diabetes management, hypertensive disorders of pregnancy, fetal monitoring, preterm birth risk, postpartum depression screening, lactation safety, and treatment-response monitoring should be evaluated with predefined escalation pathways. Studies should assess not only technical feasibility, but also false reassurance, false alarms, anxiety, clinical workload, missed events, healthcare utilization, and maternal–infant outcomes [37].

Fourth, the field should develop multimodal, context-aware, and interpretable AI that integrates wearable data with symptoms, hormone measures, CGM, EHRs, medications, environmental exposures, and patient-reported outcomes. Models should distinguish measured signals from inferred states, quantify uncertainty, and undergo drift monitoring across devices, populations, and life stages.

Fifth, reporting of women’s health digital biomarkers should be standardized. Studies should document device and firmware details, sampling and preprocessing methods, missing-data rules, reference standards, validation, outcomes, subgroup performance, intended use, and cross-device harmonization. Building on V3 and EVIDENCE, reports should also specify reproductive stage, cycle regularity, hormone therapy, pregnancy/postpartum or menopause status, symptom definitions, and hormone or ovulation reference standards [5,33].

Finally, the field must move from device validation to care-pathway studies. The key question is not whether a device can generate a signal, but whether that signal improves diagnosis, counseling, monitoring, treatment response, patient experience, access, efficiency, or clinician workload. Future studies should include patients, clinicians, caregivers, and health-system stakeholders from the beginning, so that wearable insights become actionable components of care rather than isolated data streams [38].

## 7. Conclusions

Women’s health wearables are evolving from consumer self-tracking tools into platforms for longitudinal digital phenotyping across the reproductive lifespan. Their greatest value lies in capturing dynamic physiological, behavioral, symptomatic, and functional patterns that episodic care often misses, including menstrual cyclicity, fertility-related changes, pregnancy adaptation, postpartum recovery, perimenopause, menopause, and later-life health. Yet continuous measurement alone does not create clinical value. Wearable-derived signals must be translated into biologically grounded, rigorously validated, equitable, and clinically actionable digital phenotypes.

Progress will depend on hormone-linked and multimodal longitudinal datasets, validation across diverse reproductive stages and populations, transparent and interpretable AI, standardized reporting, and prospective evaluation within care pathways. Greater attention is needed for under-measured transitions, particularly postpartum recovery, perimenopause, and menopause, where symptoms are variable, multidimensional, and closely connected to sleep, mental health, daily functioning, and quality of life. Safety-critical applications in pregnancy and postpartum care also require clearly defined oversight and escalation pathways.

For successful clinical translation, wearable outputs must provide concise, interpretable summaries that strengthen clinical judgment, patient autonomy, and shared decision-making. Supported by robust evidence, effective governance, and responsible implementation, women’s health wearables could advance more longitudinal, personalized, and prevention-oriented models of care.

## Figures and Tables

**Table 1:** Conceptual levels of wearable-derived information in women’s health.

Level	Operational Definition	Illustrative Example
**Signal**	A direct or device-derived measurement without biological or clinical interpretation	Peripheral skin temperature
**Feature**	A processed or summarized characteristic derived from one or more signals	Nocturnal temperature deviation from personal baseline
**Digital biomarker**	An objectively measured digital feature validated against an appropriate biological, physiological, or clinical reference	Validated post-ovulatory thermal shift
**Digital phenotype**	A longitudinal, context-aware integration of multiple signals and relevant biochemical, clinical, or patient-generated data representing a state or trajectory	Hormone-grounded ovulatory pattern combining temperature, LH or PdG, symptoms, and cycle timing
**Actionable summary**	A sufficiently validated and interpretable phenotype translated into a predefined response for the intended user	Summary indicating probable ovulation, confidence, and an appropriate follow-up action

**Table 3:** Operational requirements for applying the four-layer women’s health digital phenotyping framework.

Framework Layer	Representative Minimum Inputs	Key Analytical or Decision Points	Reportable Outputs	Validation or Success Criteria	Illustrative Use
**Layer 1: Data capture**	Time-stamped wearable signals; device and firmware information; sampling frequency; wear time; missingness; relevant symptoms, clinical context, or biochemical measurements	Sensor selection, signal-quality assessment, artifact removal, data harmonization, and missing-data handling	Curated longitudinal dataset with data-completeness and signal-quality indicators	Agreement with reference measures, artifact rate, wear-time adherence, missingness, and reproducibility	Collection of HRV, sleep, activity, and symptom data during postpartum recovery
**Layer 2: Physiological domain mapping**	Quality-controlled signals plus reproductive stage, symptoms, medications, and relevant biochemical or clinical context	Map signals to plausible biological domains; distinguish direct measures from proxies; assess confounders and alternative explanations	Interpretable domain-level features representing autonomic, metabolic, thermoregulatory, sleep, endocrine, mental-health, or functional processes	Physiological plausibility, association with appropriate biochemical or clinical reference standards, and consistency across relevant subgroups	Mapping temperature and hormone-linked data to probable ovulatory physiology
**Layer 3: Computational phenotyping**	Domain-level features, personalized baselines, observation windows, relevant labels or outcomes, and data-quality indicators	Selection of rule-based, statistical, or AI methods; temporal modeling; multimodal fusion; threshold definition; uncertainty estimation	Phenotype, trajectory, forecast, or risk estimate with confidence, observation window, and contributing signals	Sensitivity, specificity, discrimination, calibration, agreement, robustness to missing data, subgroup performance, and external validation	Identification of a persistent postpartum recovery-mismatch phenotype
**Layer 4: Actionable translation**	Sufficiently validated phenotype, intended user, clinical context, decision threshold, and defined care pathway	Selection of response level, human oversight, escalation responsibility, alert management, and communication format	Patient-facing or clinician-facing summary with uncertainty and a predefined response, such as self-management, routine review, or escalation	Clinical utility, false-alert burden, time to appropriate action, workflow impact, safety, usability, and patient-centered outcomes	Earlier postpartum review when persistent multimodal deterioration is detected

## Data Availability

Data sharing is not applicable, as this research did not generate any new data.

## List of Abbreviations

ACOG	American College of Obstetricians and Gynecologists
AI	Artificial Intelligence
BMI	Body Mass Index
CGM	Continuous Glucose Monitoring
E3G	Estrone-3-Glucuronide
ECG	Electrocardiogram
EHR	Electronic Health Record
EVIDENCE	EVIDENCE Publication Checklist for Studies Evaluating Connected Sensor Technologies
FDA	U.S. Food and Drug Administration
FSH	Follicle-Stimulating Hormone
HbA1c	Hemoglobin A1c
HR	Heart Rate
HRV	Heart Rate Variability
LH	Luteinizing Hormone
NIST	National Institute of Standards and Technology
OGTT	Oral Glucose Tolerance Test
PdG	Pregnanediol Glucuronide
PMOS	Polyendocrine Metabolic Ovarian Syndrome
RMSSD	Root Mean Square of Successive Differences
V3	Verification, Analytical Validation, and Clinical Validation
